# Supplementary material for: Back-projection improves inference from sparsely sampled genomic surveillance data
Source: bioRxiv. 2025 Jul 1:2025.06.29.662219. Preprint. [Version 1] doi: 10.1101/2025.06.29.662219 (PMC12236715; doi:10.1101/2025.06.29.662219)
Supplement: Supplement 1 [file NIHPP2025.06.29.662219v1-supplement-1.pdf]

bioRxiv preprint doi: https://doi.org/10.1101/2025.06.29.662219; this version posted July 1, 2025. The copyright holder for this preprint (which was not certified by peer review) is the author/funder, who has granted bioRxiv a license to display the preprint in perpetuity. It is made available under aCC-BY 4.0 International license.

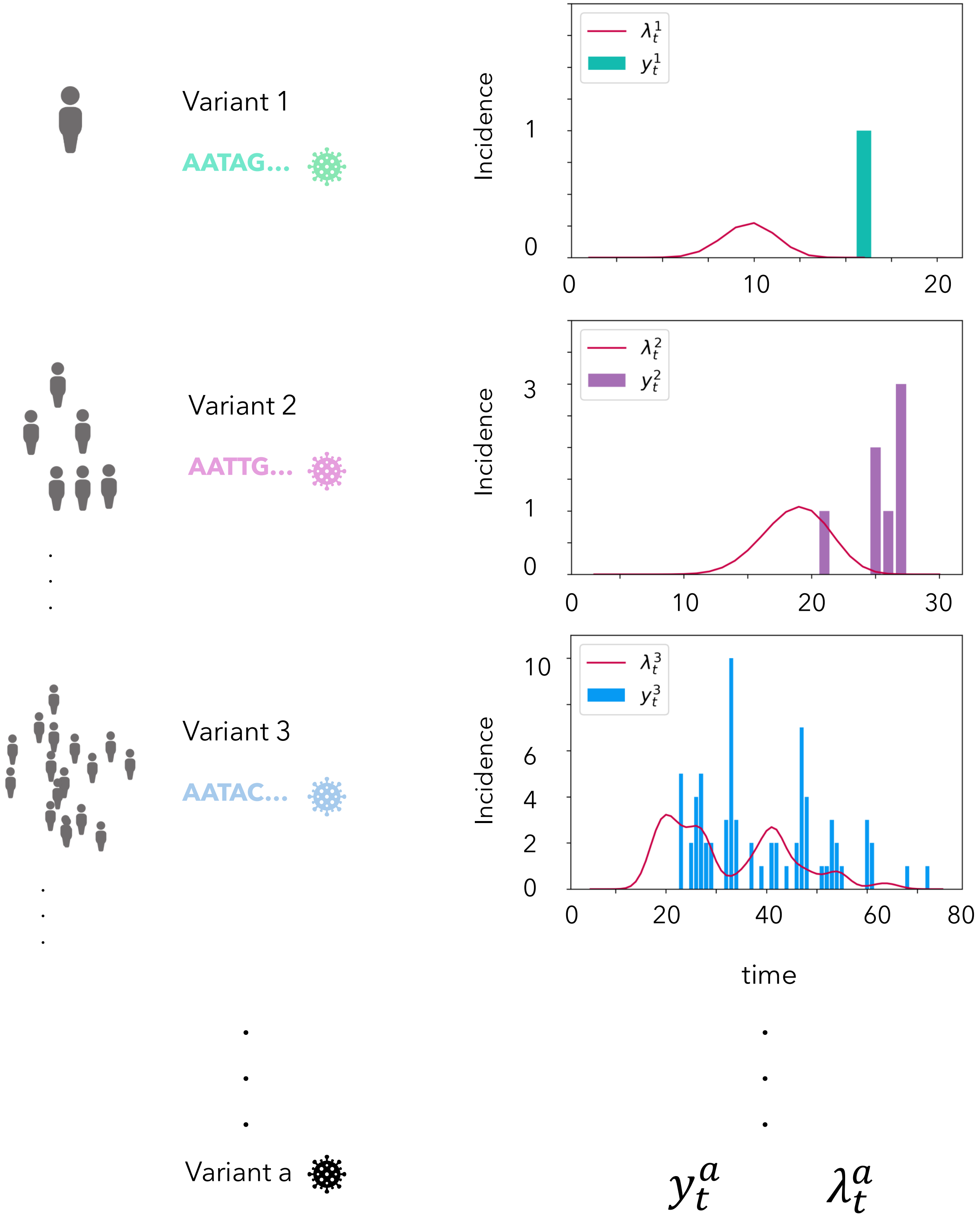

Supplementary Fig. 1. Dividing up sequences for back-projection processing, graphical illustration The SARS-CoV-2 genome is of size 30kbp (kilo base pairs). For a viral genome this large, some SNV (single nucleotide variant) mutations only appear in combination once in a regional outbreak. Other SNVs in the same combination spread to multiple people during an outbreak. We group individuals passing along the same genetic variation of SARS-CoV-2 before smoothing these sequence counts via back-projection. We perform this division as a pre-processing step to allow us to replace integer value sequence counts in our additive model of evolution and selection inference, with normalized non-integer weights of counts at a given discrete time point. Each SNV, a, is separated into its own population of observed cases, y_t^a, with which we estimate the incidence λ_t^a (Methods). In both our original method and our implementation of back-projection, these "populations" of unique and non-unique sequences have their alleles combined with an additive model, in order to create single allele frequencies which make up that allele's evolutionary history.

bioRxiv preprint doi: https://doi.org/10.1101/2025.06.29.662219; this version posted July 1, 2025. The copyright holder for this preprint (which was not certified by peer review) is the author/funder, who has granted bioRxiv a license to display the preprint in perpetuity. It is made available under aCC-BY 4.0 International license.

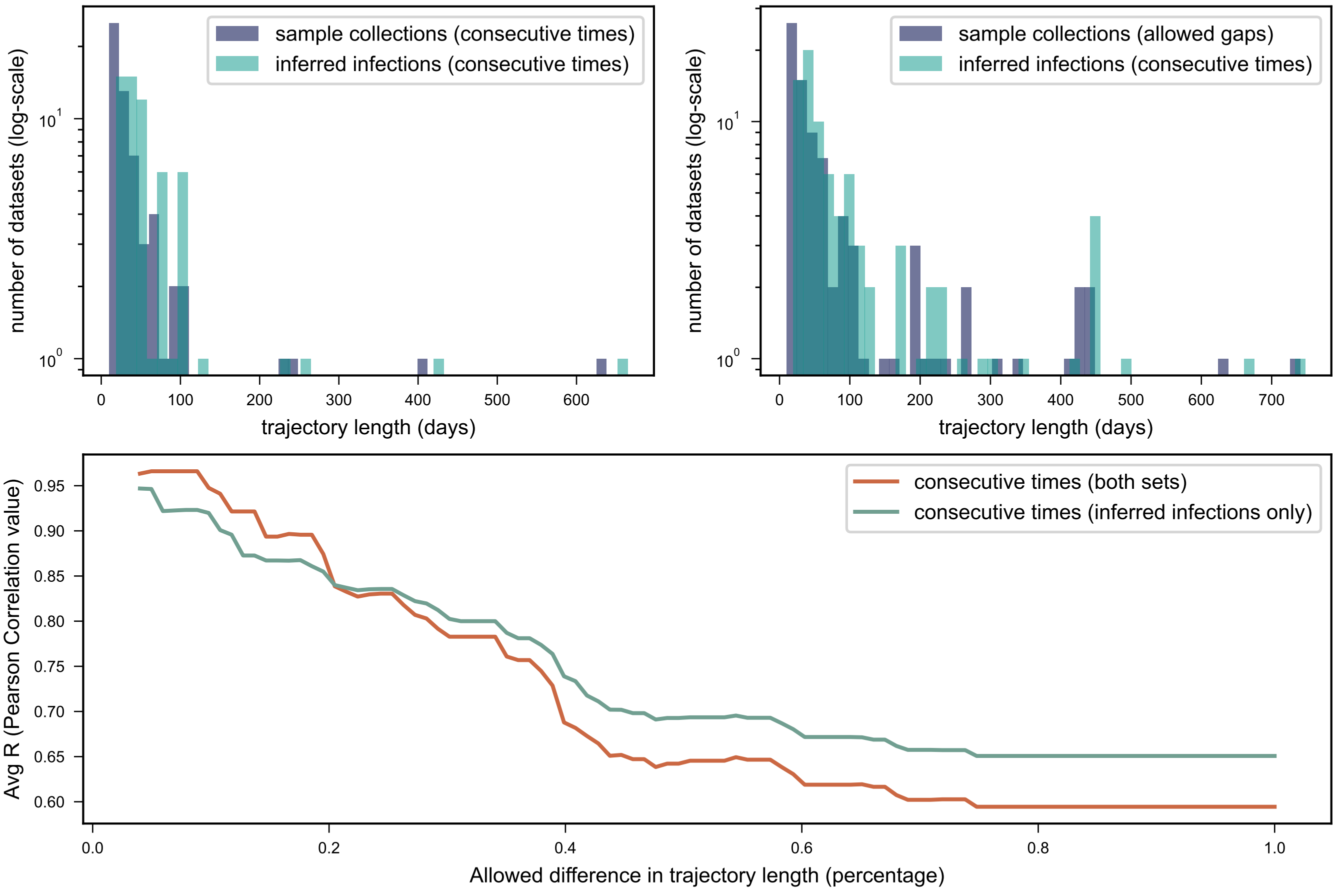

Supplementary Fig. 2. Pearson's correlations at different trajectory length thresholds As a result of back projection increasing trajectory length and providing smoothed estimates over data gaps, the correlations we obtain when we compare the inferred selection effects of back projected inferred infections, and unprocessed sample collections, can vary. In particular, the difference in lengths between the starting trajectory and the back-projected trajectory can have an effect on the Pearson correlation values that we calculate. Figure **a.** and figure **b.** show log-scaled histograms of the length of trajectories, in both a set of completely contiguous trajectories for both data, and a set where back-projected estimates have contiguous data, but we allow for the sample collections to contain data gaps. For both sets, we calculate the average Pearson Correlation based on a threshold which subsets the allowed trajectories to be averaged, based on how dissimilar the length of the inferred infection trajectories are from the sample collection trajectories. We obtain good correlation (0.97-0.85) for paired trajectory lengths that do not deviate by more than 20 %. From there, as the length of the inferred infection trajectories becomes more dissimilar to the sample collection trajectories, the average correlation falls off.

bioRxiv preprint doi: https://doi.org/10.1101/2025.06.29.662219; this version posted July 1, 2025. The copyright holder for this preprint (which was not certified by peer review) is the author/funder, who has granted bioRxiv a license to display the preprint in perpetuity. It is made available under aCC-BY 4.0 International license.

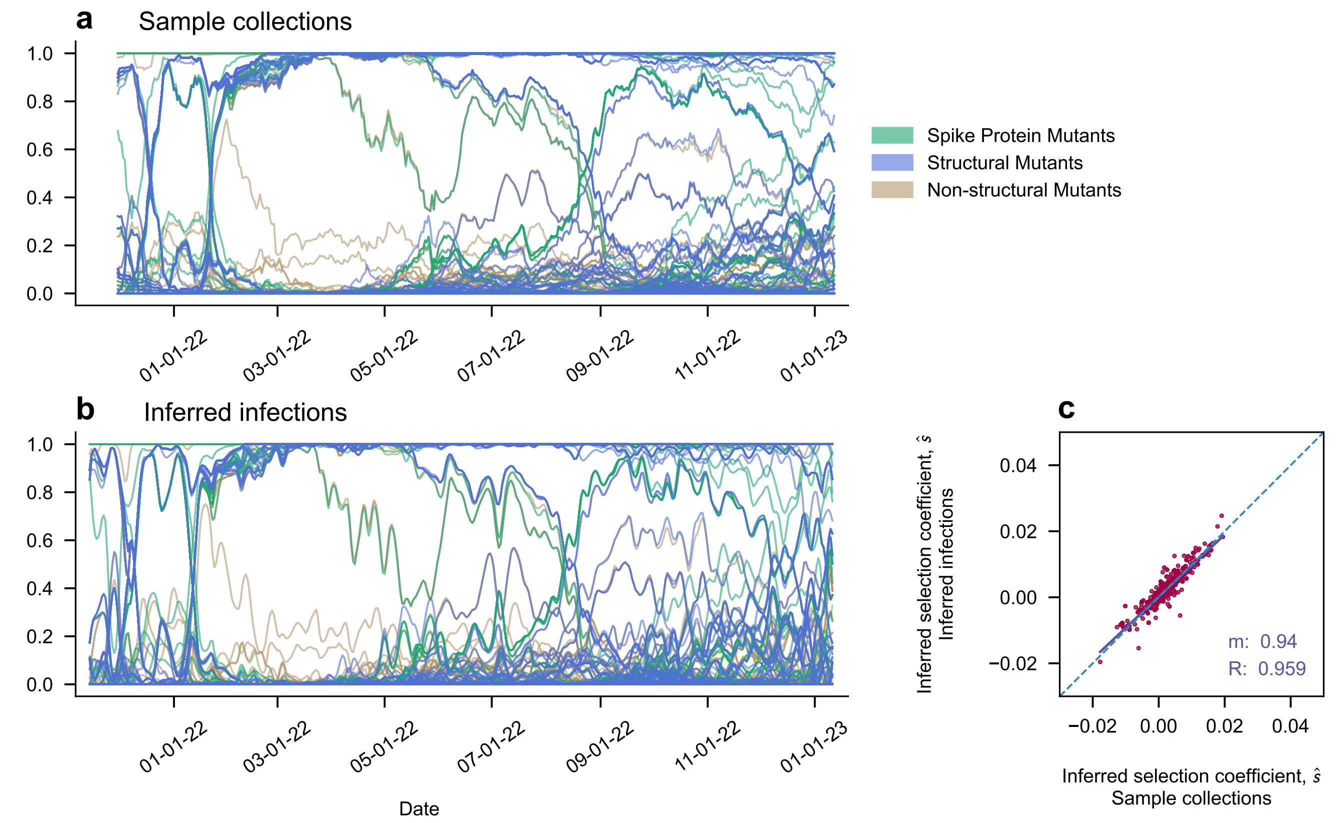

Supplementary Fig. 3. Selection coefficients with and without back-projection are highly correlated in long trajectories (Hong Kong). Trajectories across long time periods show smoother dynamics, with large agreement.

bioRxiv preprint doi: https://doi.org/10.1101/2025.06.29.662219; this version posted July 1, 2025. The copyright holder for this preprint (which was not certified by peer review) is the author/funder, who has granted bioRxiv a license to display the preprint in perpetuity. It is made available under aCC-BY 4.0 International license.

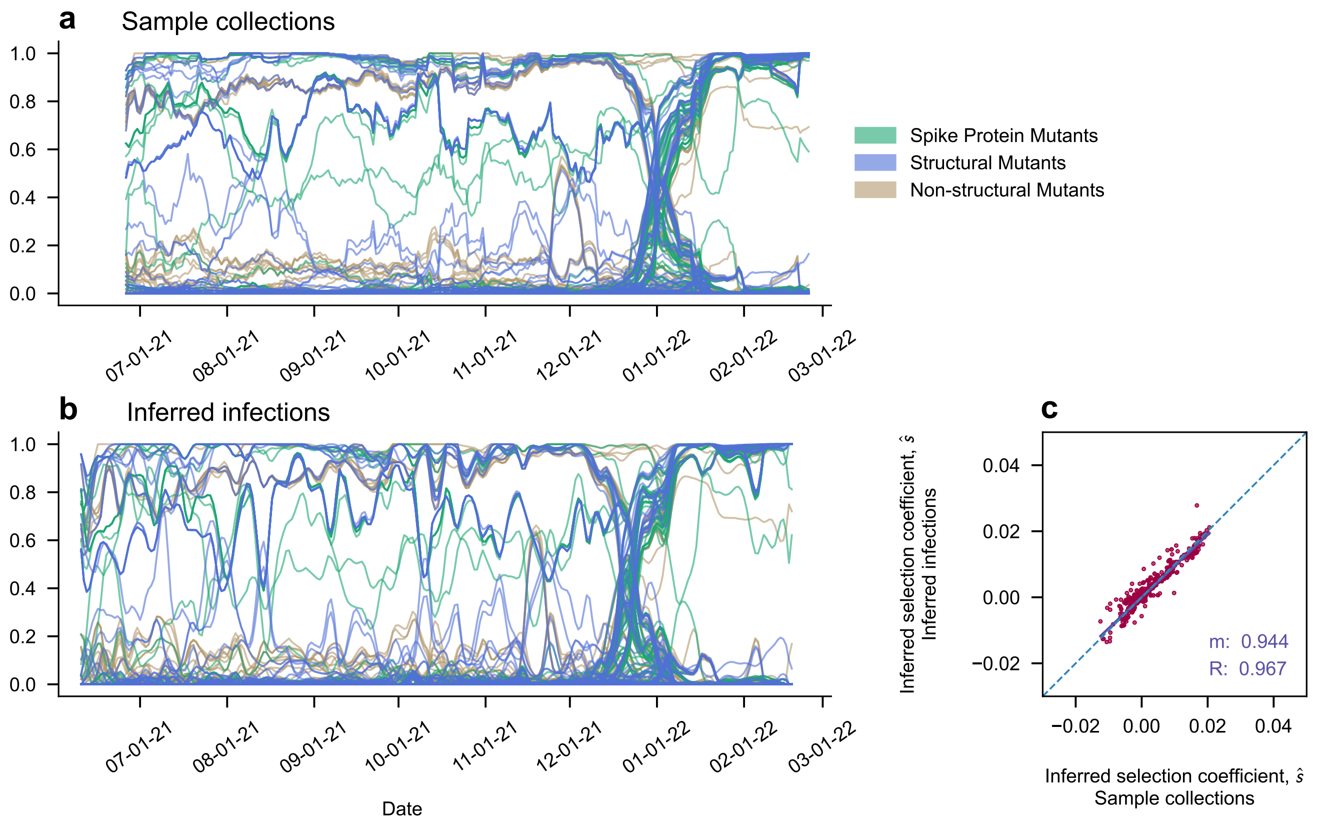

Supplementary Fig. 4. Selection coefficients with and without back-projection are highly correlated in long trajectories (Karnataka). Trajectories across long time periods show smoother dynamics, with large agreement.

bioRxiv preprint doi: https://doi.org/10.1101/2025.06.29.662219; this version posted July 1, 2025. The copyright holder for this preprint (which was not certified by peer review) is the author/funder, who has granted bioRxiv a license to display the preprint in perpetuity. It is made available under aCC-BY 4.0 International license.

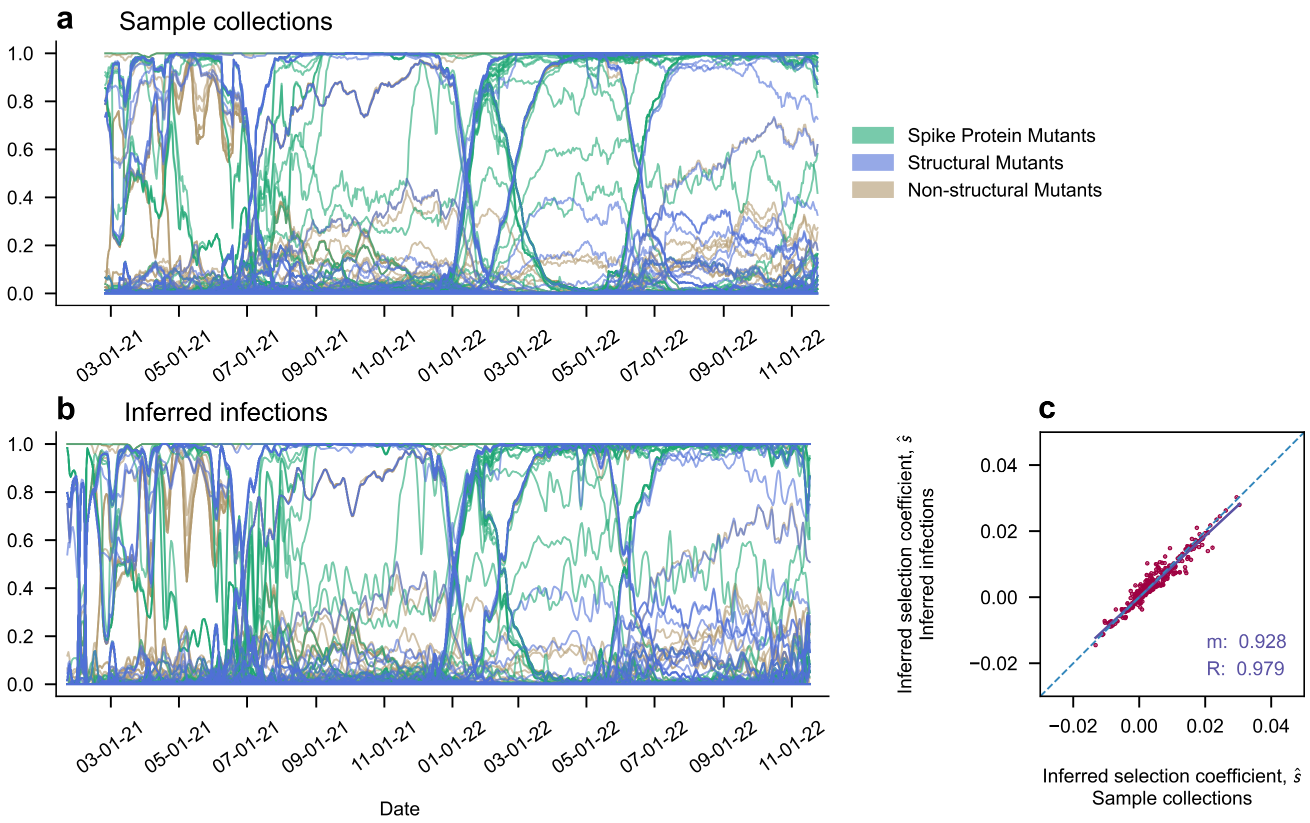

Supplementary Fig. 5. Selection coefficients with and without back-projection are highly correlated in long trajectories (Slovakia). Trajectories across long time periods show smoother dynamics, with large agreement.

bioRxiv preprint doi: https://doi.org/10.1101/2025.06.29.662219; this version posted July 1, 2025. The copyright holder for this preprint (which was not certified by peer review) is the author/funder, who has granted bioRxiv a license to display the preprint in perpetuity. It is made available under aCC-BY 4.0 International license.

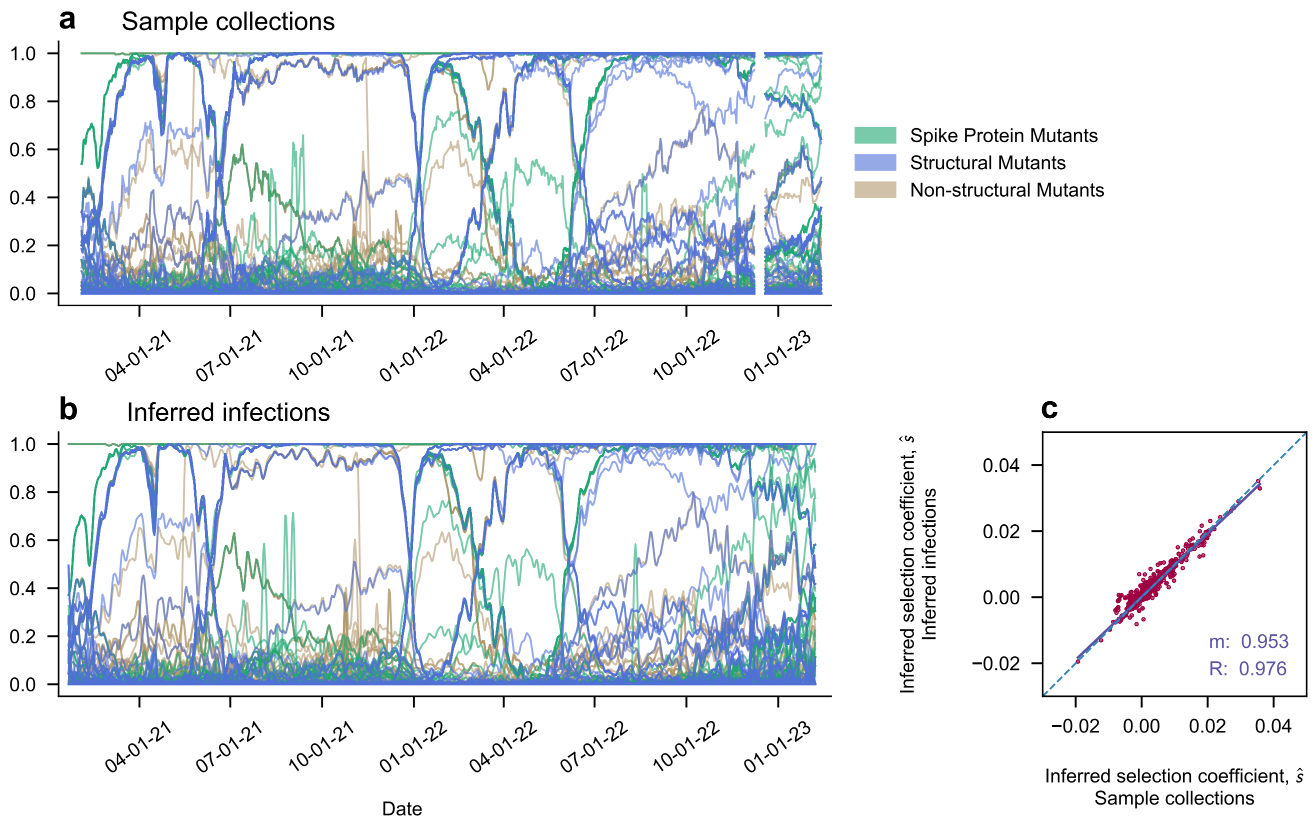

Supplementary Fig. 6. Selection coefficients with and without back-projection are highly correlated in long trajectories (Croatia). Trajectories across long time periods show smoother dynamics, with agreement even allowing for small gaps in the unprocessed frame.

bioRxiv preprint doi: https://doi.org/10.1101/2025.06.29.662219; this version posted July 1, 2025. The copyright holder for this preprint (which was not certified by peer review) is the author/funder, who has granted bioRxiv a license to display the preprint in perpetuity. It is made available under aCC-BY 4.0 International license.

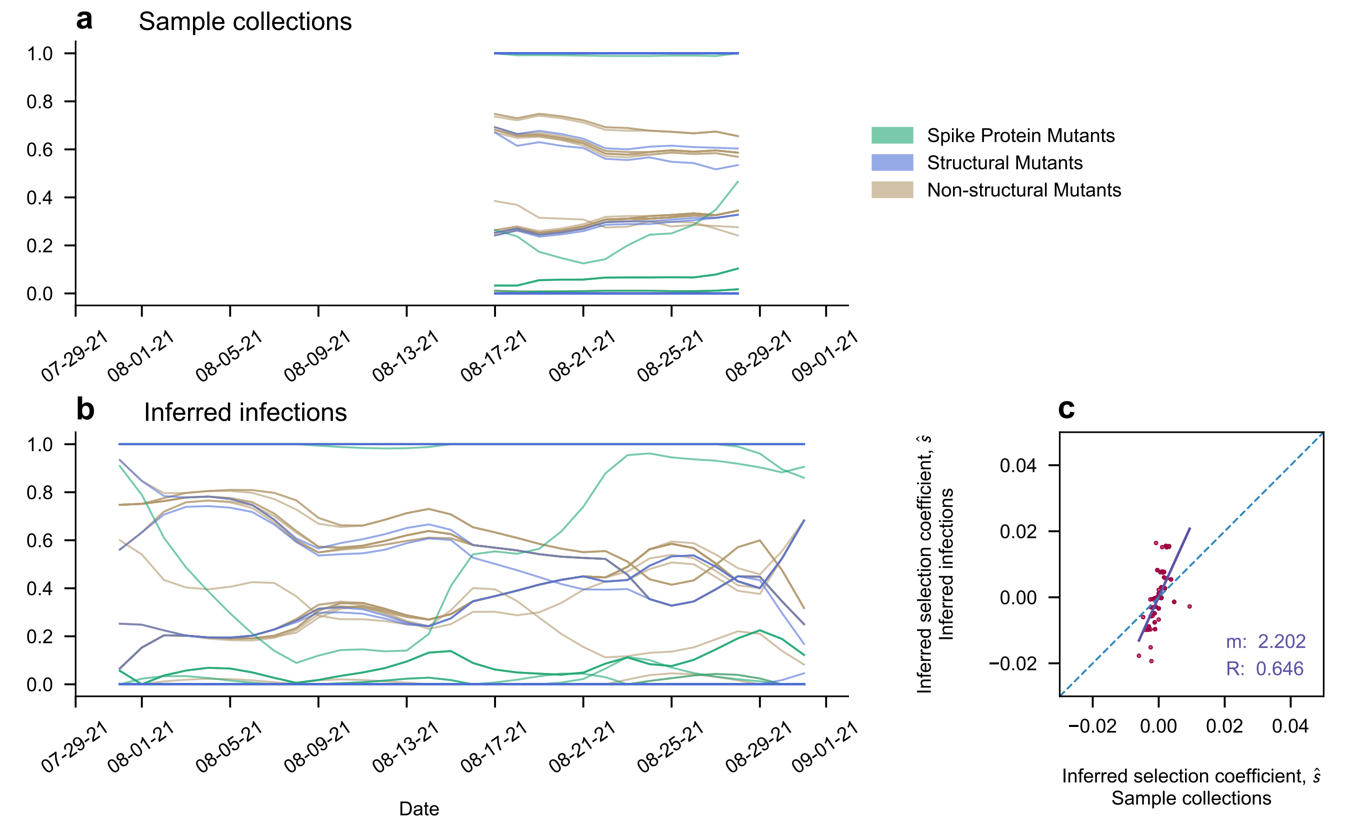

Supplementary Fig. 7. Correlated effects can vary when comparing trajectories of different lengths (Kenya). Back-projected estimates often provide a greater amount of trajectory information

bioRxiv preprint doi: https://doi.org/10.1101/2025.06.29.662219; this version posted July 1, 2025. The copyright holder for this preprint (which was not certified by peer review) is the author/funder, who has granted bioRxiv a license to display the preprint in perpetuity. It is made available under aCC-BY 4.0 International license.

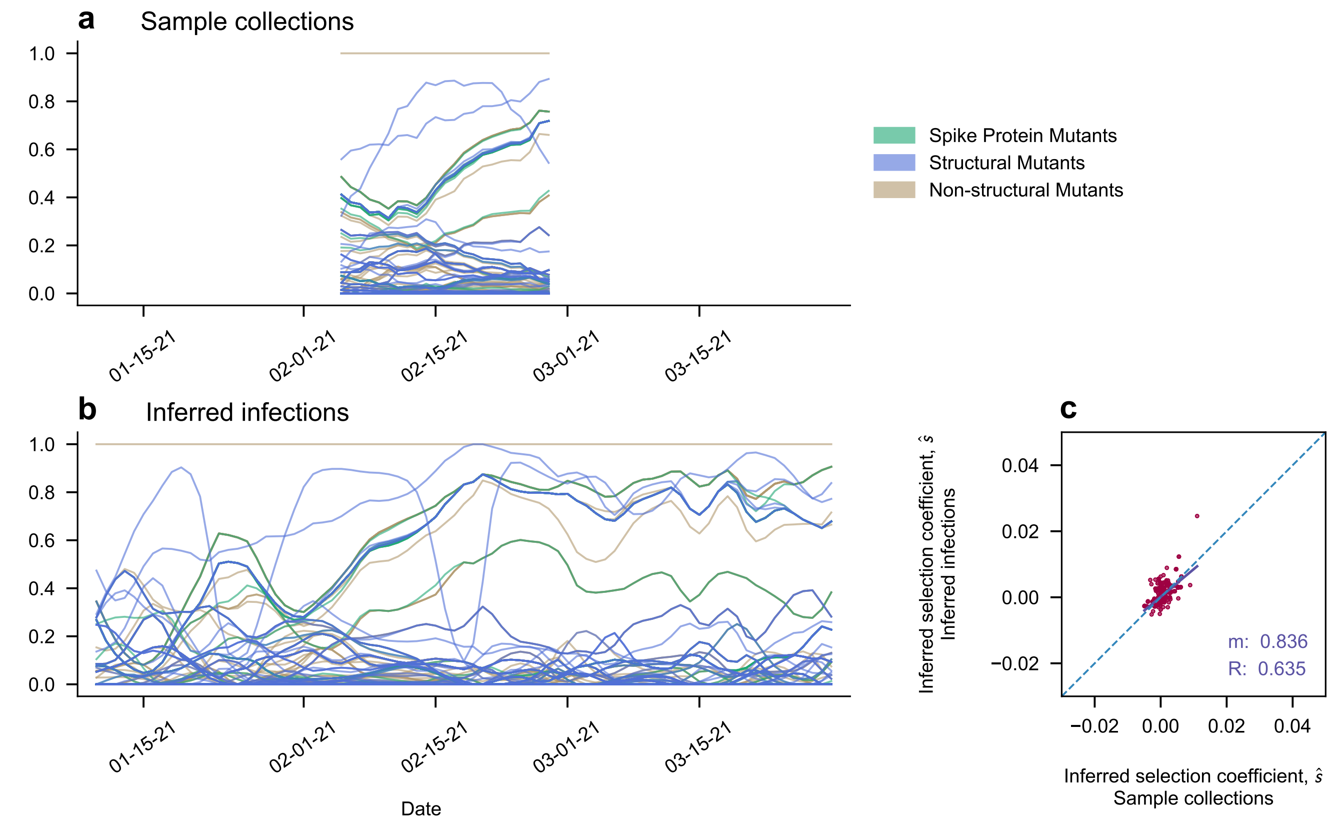

Supplementary Fig. 8. Correlated effects can vary when comparing trajectories of different lengths (Reunion). Back-projected estimates often provide a greater amount of trajectory information

bioRxiv preprint doi: https://doi.org/10.1101/2025.06.29.662219; this version posted July 1, 2025. The copyright holder for this preprint (which was not certified by peer review) is the author/funder, who has granted bioRxiv a license to display the preprint in perpetuity. It is made available under aCC-BY 4.0 International license.

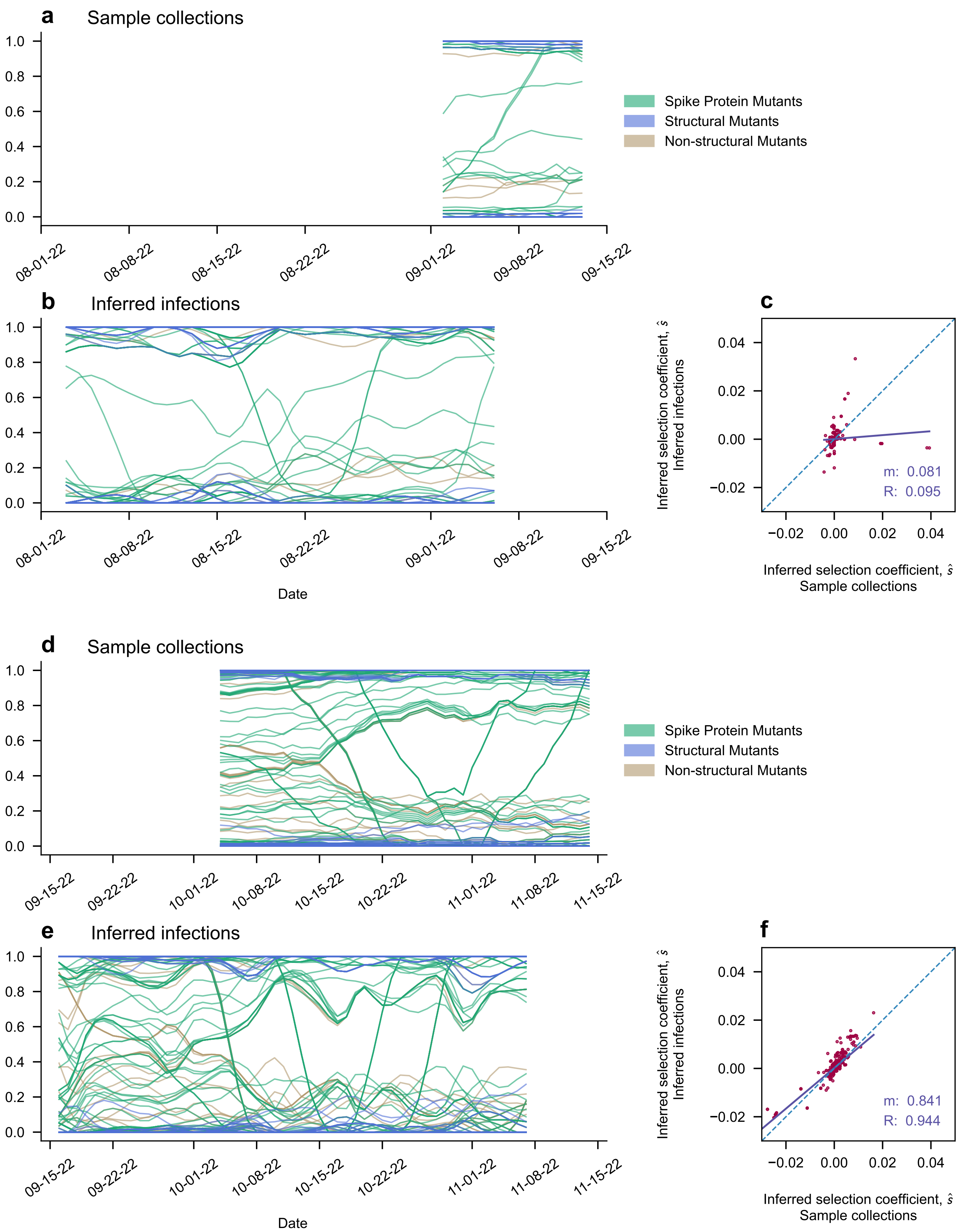

Supplementary Fig. 9. Correlated effects can vary when comparing trajectories of different lengths (Gujarat). Back Projected estimates often provide a greater amount of trajectory information. Here, the displacement of Omicron BA.1 by more transmissible variants such as BA.2, BM.1, BA.5, BQ.1 and XBB.1 cause sweeps of Spike protein mutations.

bioRxiv preprint doi: https://doi.org/10.1101/2025.06.29.662219; this version posted July 1, 2025. The copyright holder for this preprint (which was not certified by peer review) is the author/funder, who has granted bioRxiv a license to display the preprint in perpetuity. It is made available under aCC-BY 4.0 International license.

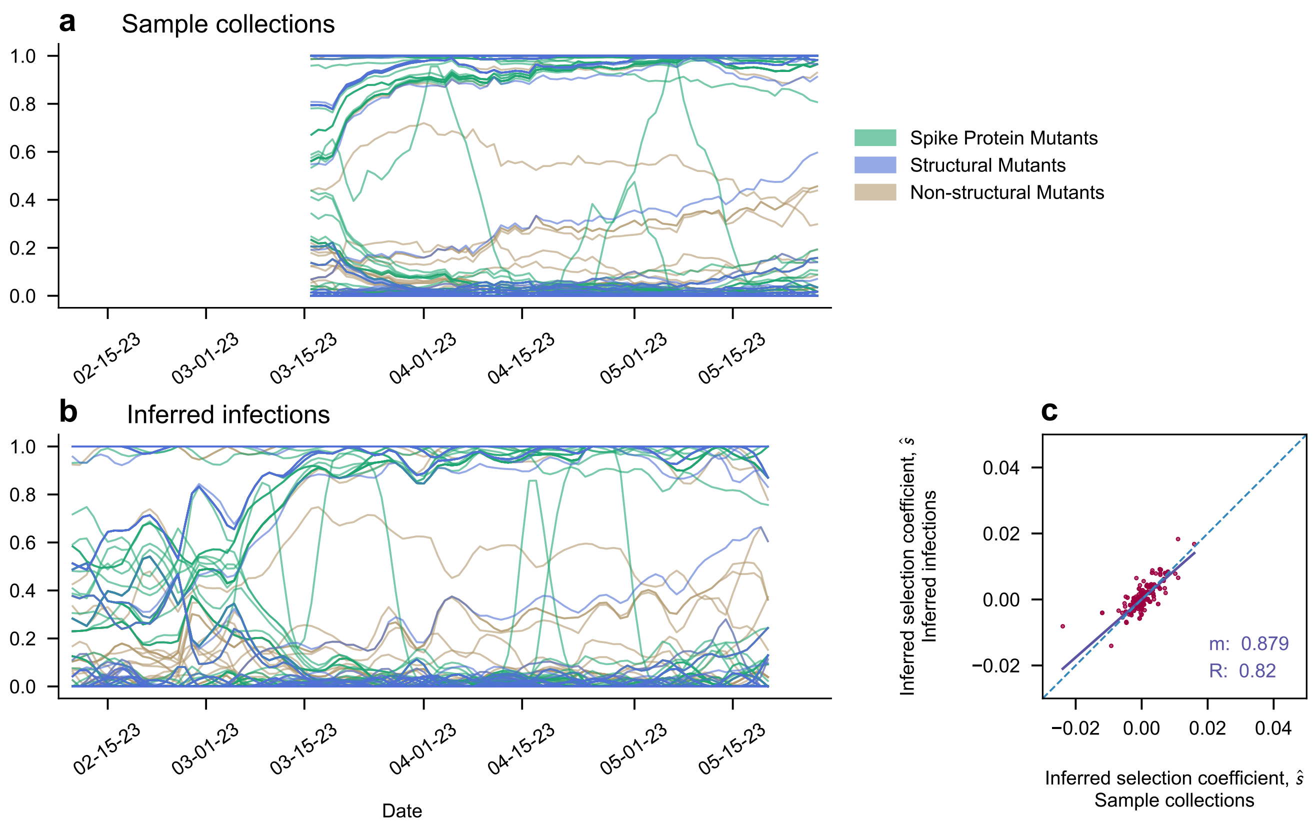

Supplementary Fig. 10. Correlated effects can vary when comparing trajectories of different lengths (Croatia). Back Projected estimates often provide a greater amount of trajectory information. Here, the displacement of Omicron BA.1 by more transmissible variants such as BA.5, BQ.1 and XBB.1 cause sweeps of Spike protein mutations.[68]

bioRxiv preprint doi: https://doi.org/10.1101/2025.06.29.662219; this version posted July 1, 2025. The copyright holder for this preprint (which was not certified by peer review) is the author/funder, who has granted bioRxiv a license to display the preprint in perpetuity. It is made available under aCC-BY 4.0 International license.

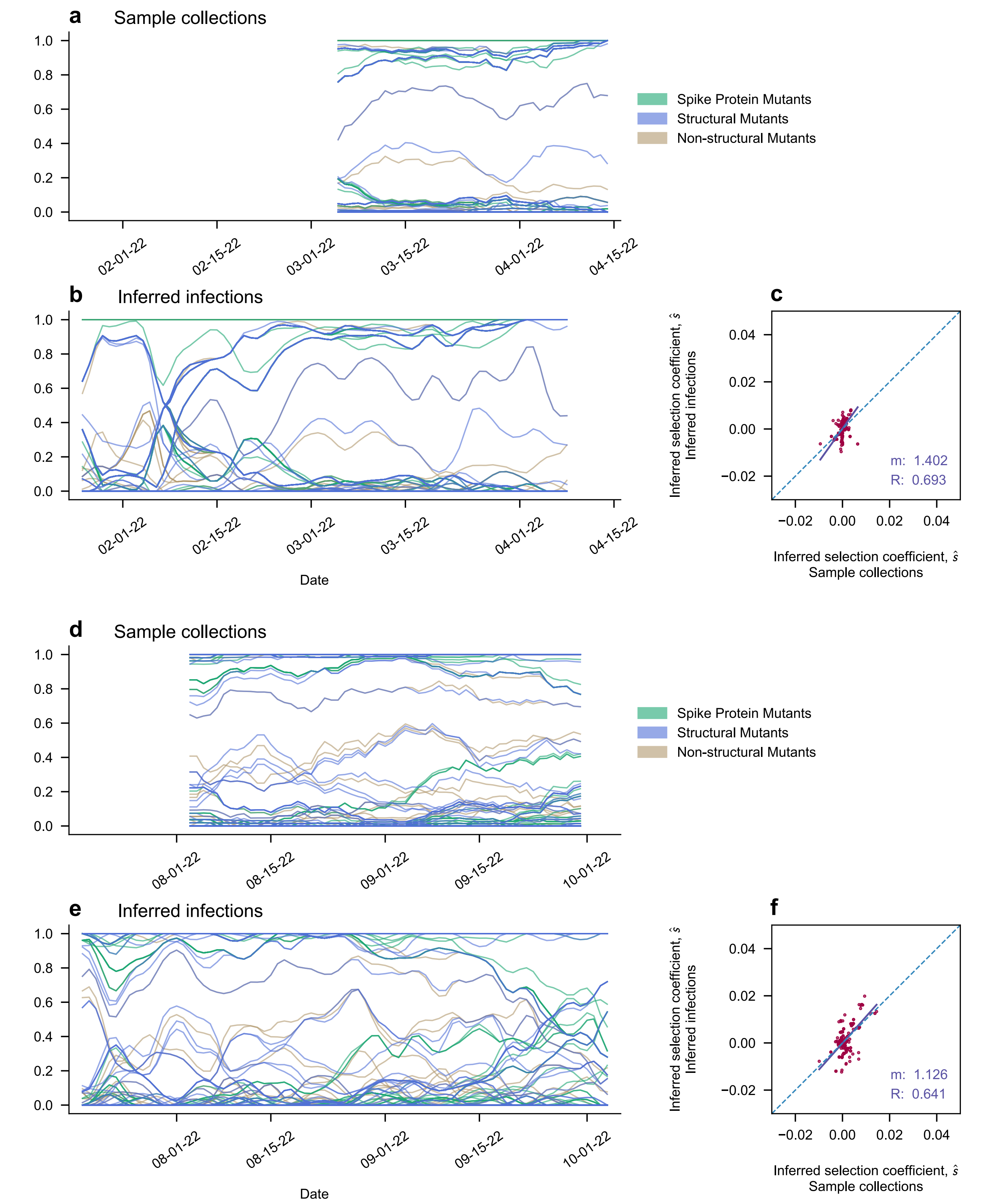

Supplementary Fig. 11. Outbreak: Vietnam Trajectory dynamics throughout waves of Omicron in Vietnam. Omicron appeared in Vietnam in early 2022[69] and by late 2022 had many circulating variants of Omicron subtypes.[70]

bioRxiv preprint doi: https://doi.org/10.1101/2025.06.29.662219; this version posted July 1, 2025. The copyright holder for this preprint (which was not certified by peer review) is the author/funder, who has granted bioRxiv a license to display the preprint in perpetuity. It is made available under aCC-BY 4.0 International license.

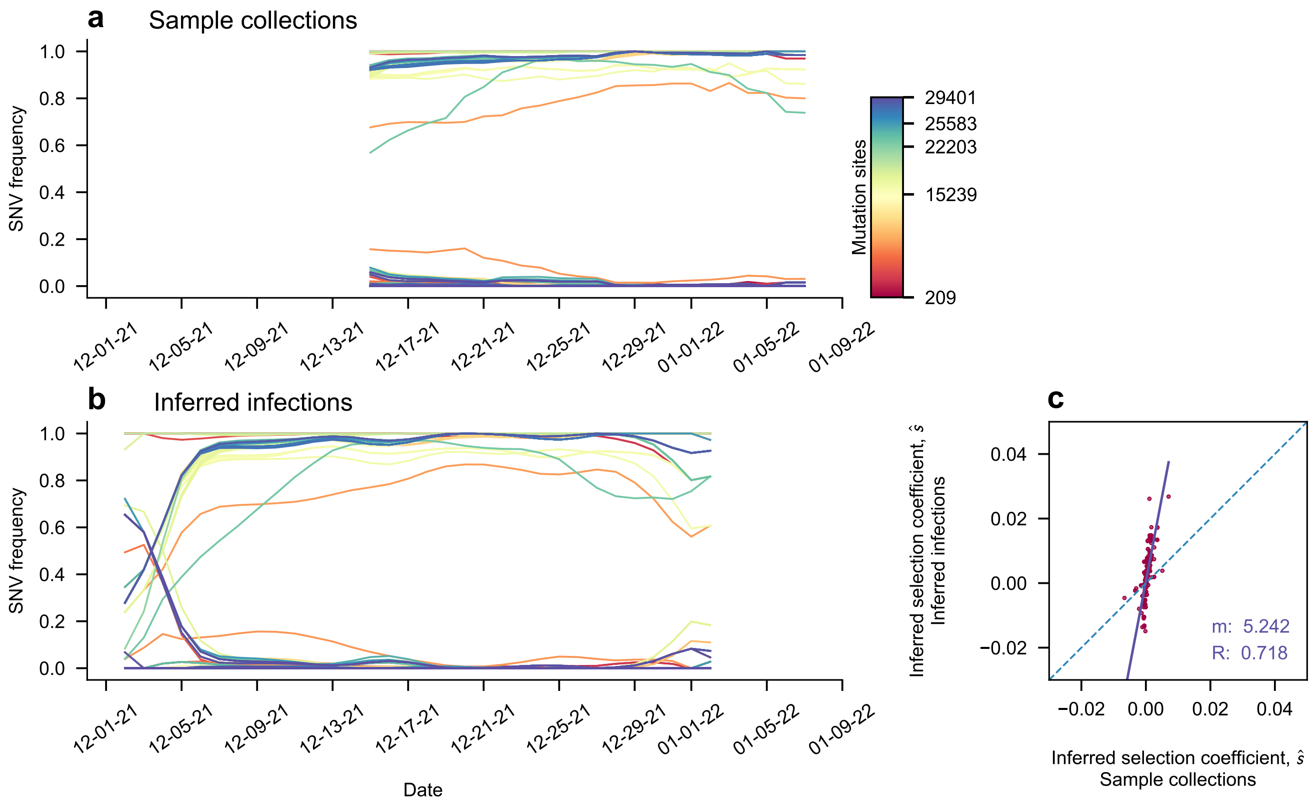

Supplementary Fig. 12. Missed rise of Omicron sweep in multiple regions (Kenya). Some regions contain poor sampling before the Omicron BA.1 rise. Back-projecting trajectory information provides additional information to infer stronger selection coefficients.

bioRxiv preprint doi: https://doi.org/10.1101/2025.06.29.662219; this version posted July 1, 2025. The copyright holder for this preprint (which was not certified by peer review) is the author/funder, who has granted bioRxiv a license to display the preprint in perpetuity. It is made available under aCC-BY 4.0 International license.

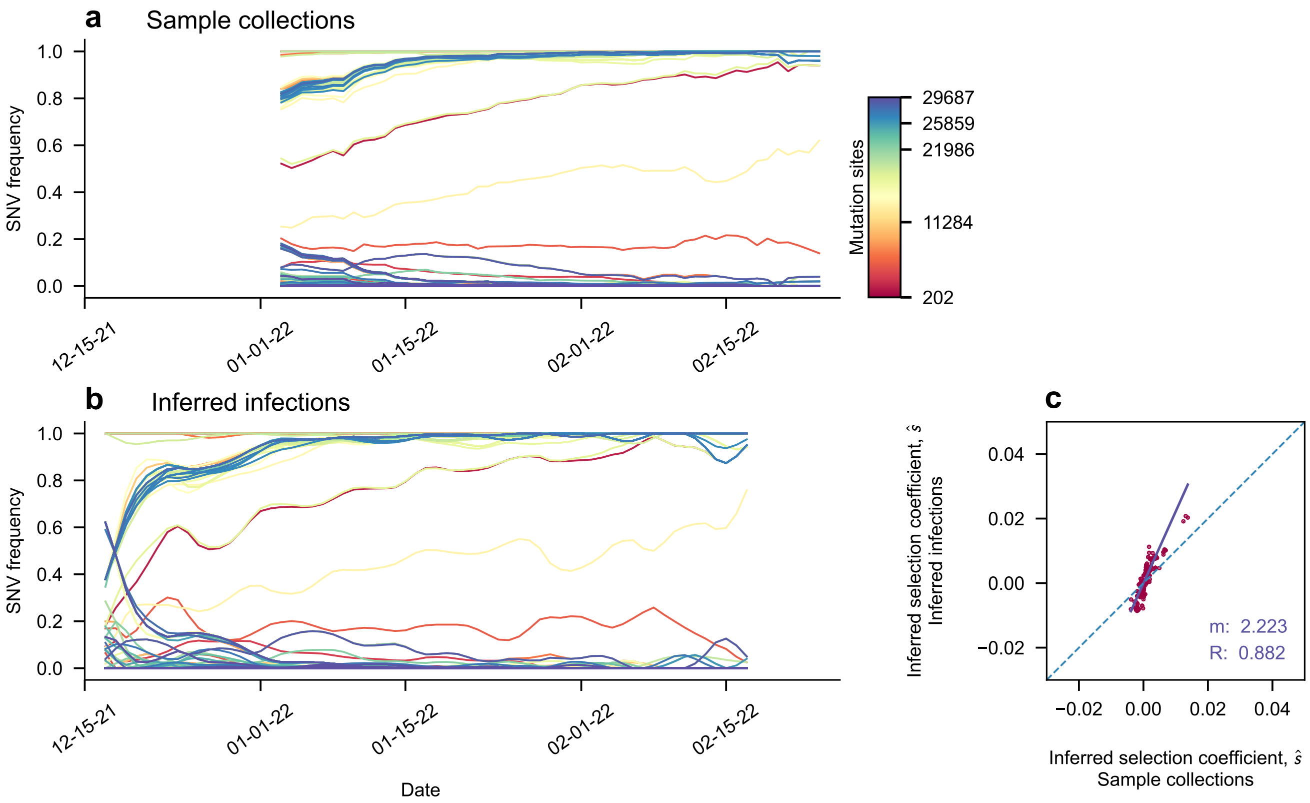

Supplementary Fig. 13. Missed rise of Omicron sweep in multiple regions (Wyoming). Some regions contain poor sampling before the Omicron BA.1 rise. Back-projecting trajectory information provides additional information to infer stronger selection coefficients.

bioRxiv preprint doi: https://doi.org/10.1101/2025.06.29.662219; this version posted July 1, 2025. The copyright holder for this preprint (which was not certified by peer review) is the author/funder, who has granted bioRxiv a license to display the preprint in perpetuity. It is made available under aCC-BY 4.0 International license.

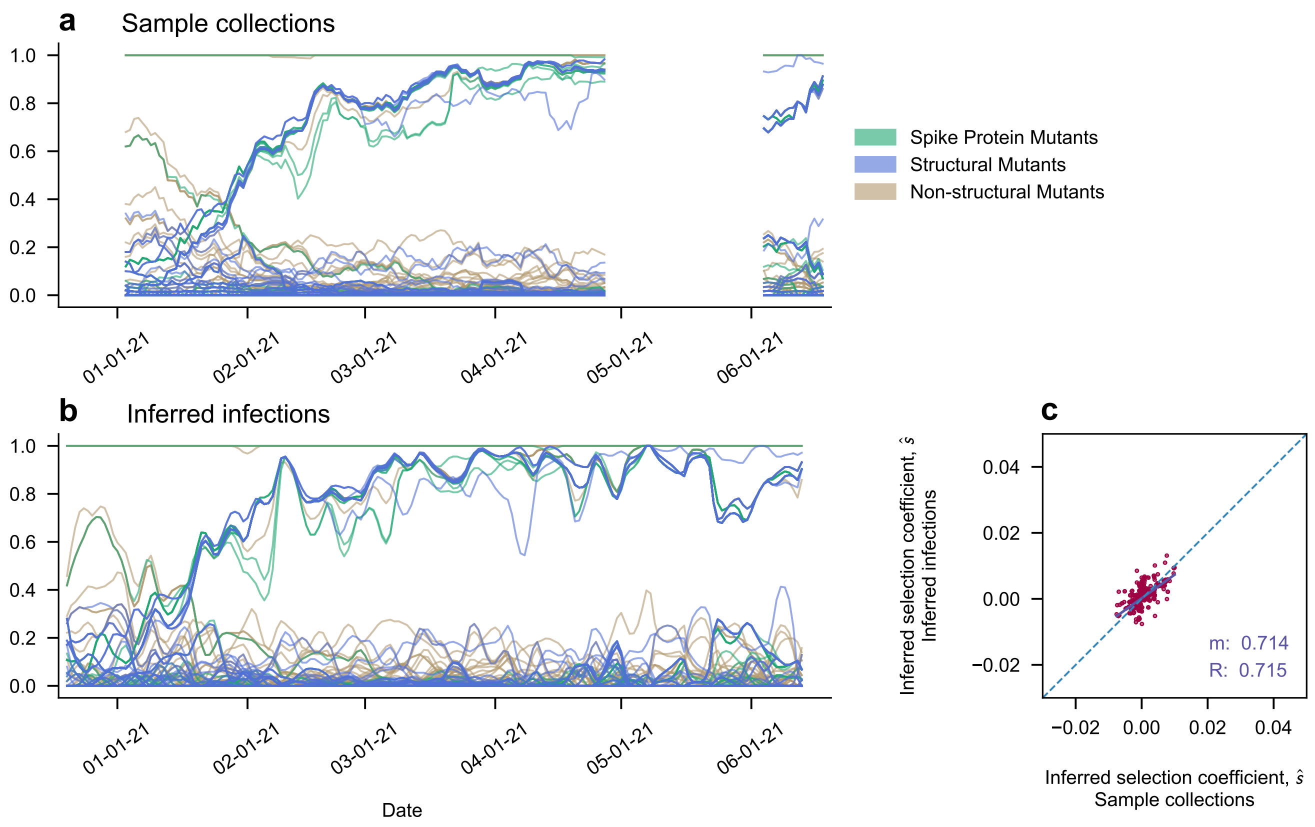

Supplementary Fig. 14. Back-projected estimates smooth over gaps in data (Romania). Back-projected trajectories fill in gaps in data.

bioRxiv preprint doi: https://doi.org/10.1101/2025.06.29.662219; this version posted July 1, 2025. The copyright holder for this preprint (which was not certified by peer review) is the author/funder, who has granted bioRxiv a license to display the preprint in perpetuity. It is made available under aCC-BY 4.0 International license.

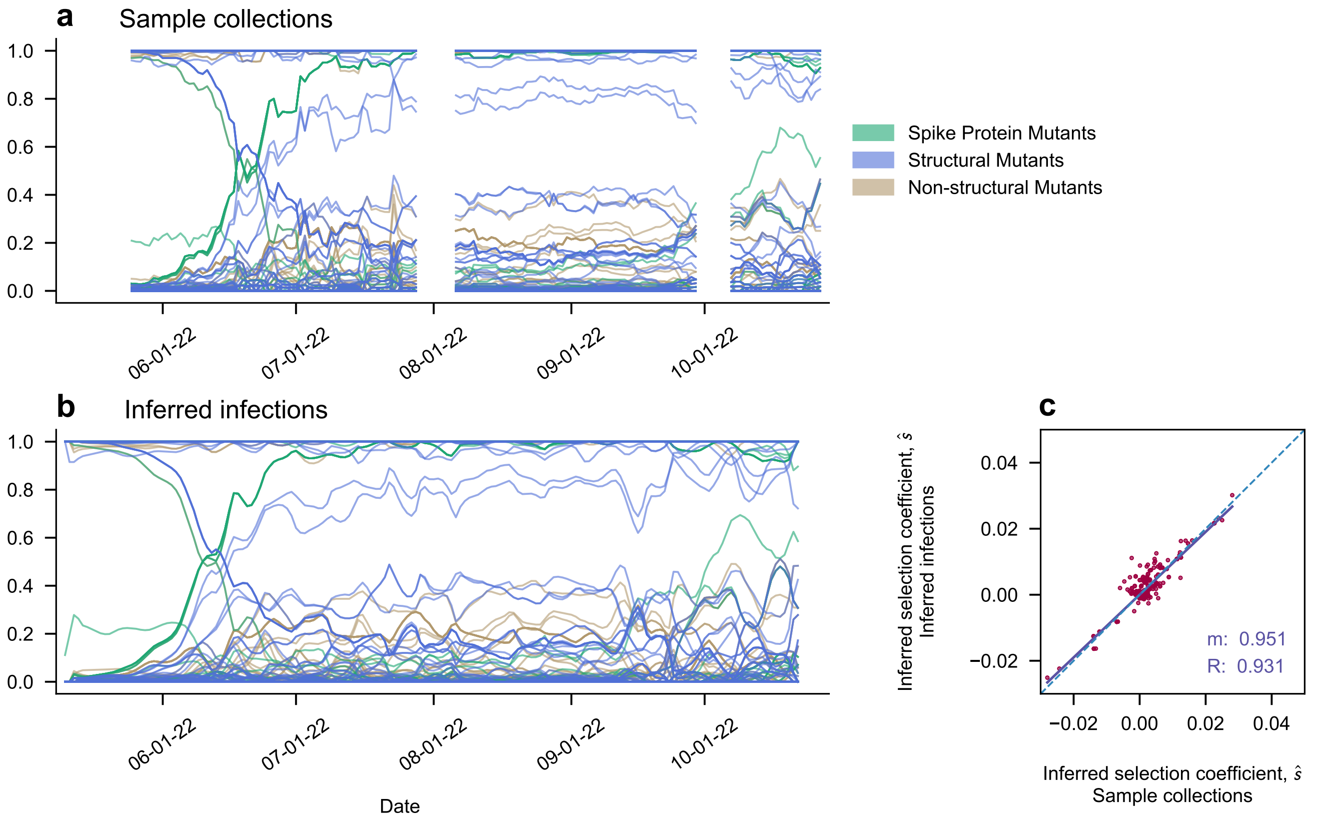

Supplementary Fig. 15. Back-projected estimates smooth over gaps in data (Reunion). Inferred trajectories fill in gaps in data.

bioRxiv preprint doi: https://doi.org/10.1101/2025.06.29.662219; this version posted July 1, 2025. The copyright holder for this preprint (which was not certified by peer review) is the author/funder, who has granted bioRxiv a license to display the preprint in perpetuity. It is made available under aCC-BY 4.0 International license.

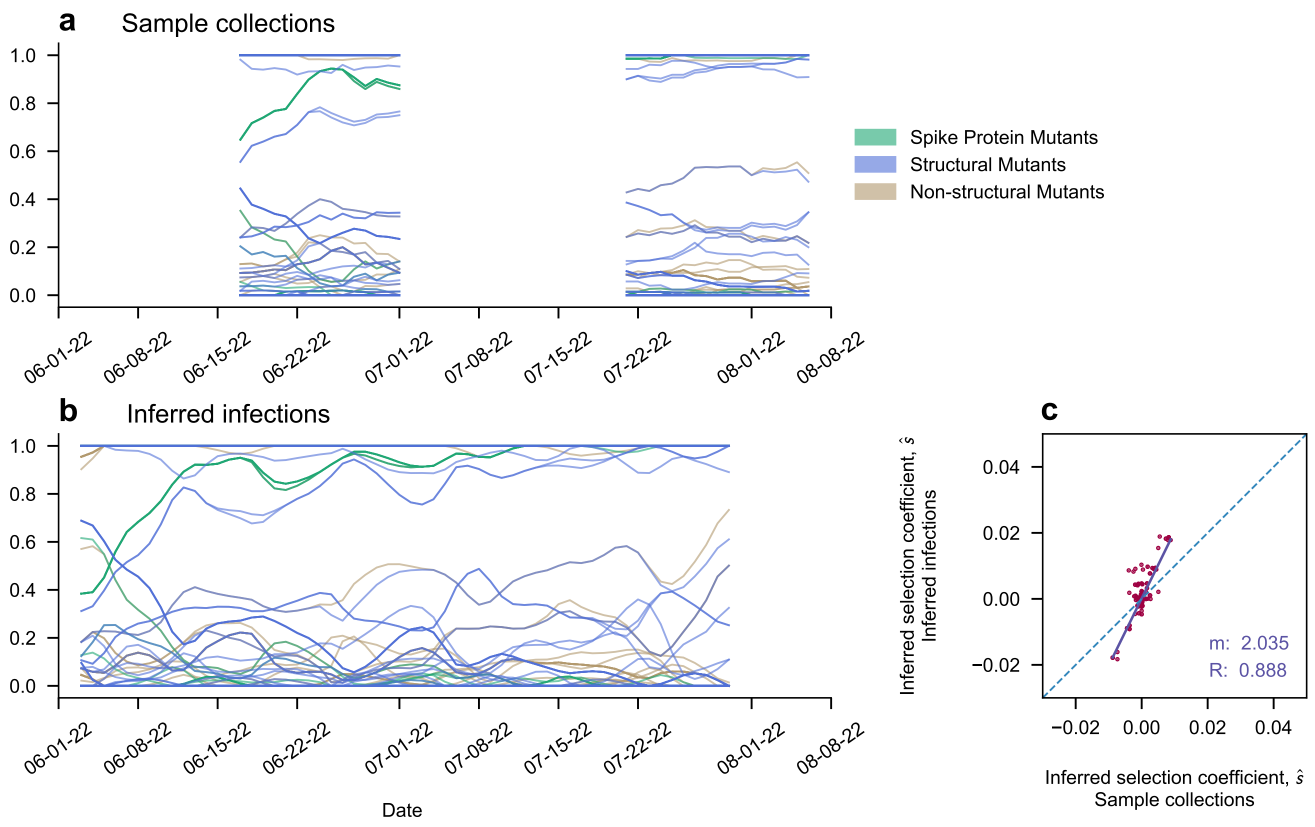

Supplementary Fig. 16. Back-projected estimates smooth over gaps in data (Cyprus). Inferred trajectories fill in gaps in data.

bioRxiv preprint doi: https://doi.org/10.1101/2025.06.29.662219; this version posted July 1, 2025. The copyright holder for this preprint (which was not certified by peer review) is the author/funder, who has granted bioRxiv a license to display the preprint in perpetuity. It is made available under aCC-BY 4.0 International license.

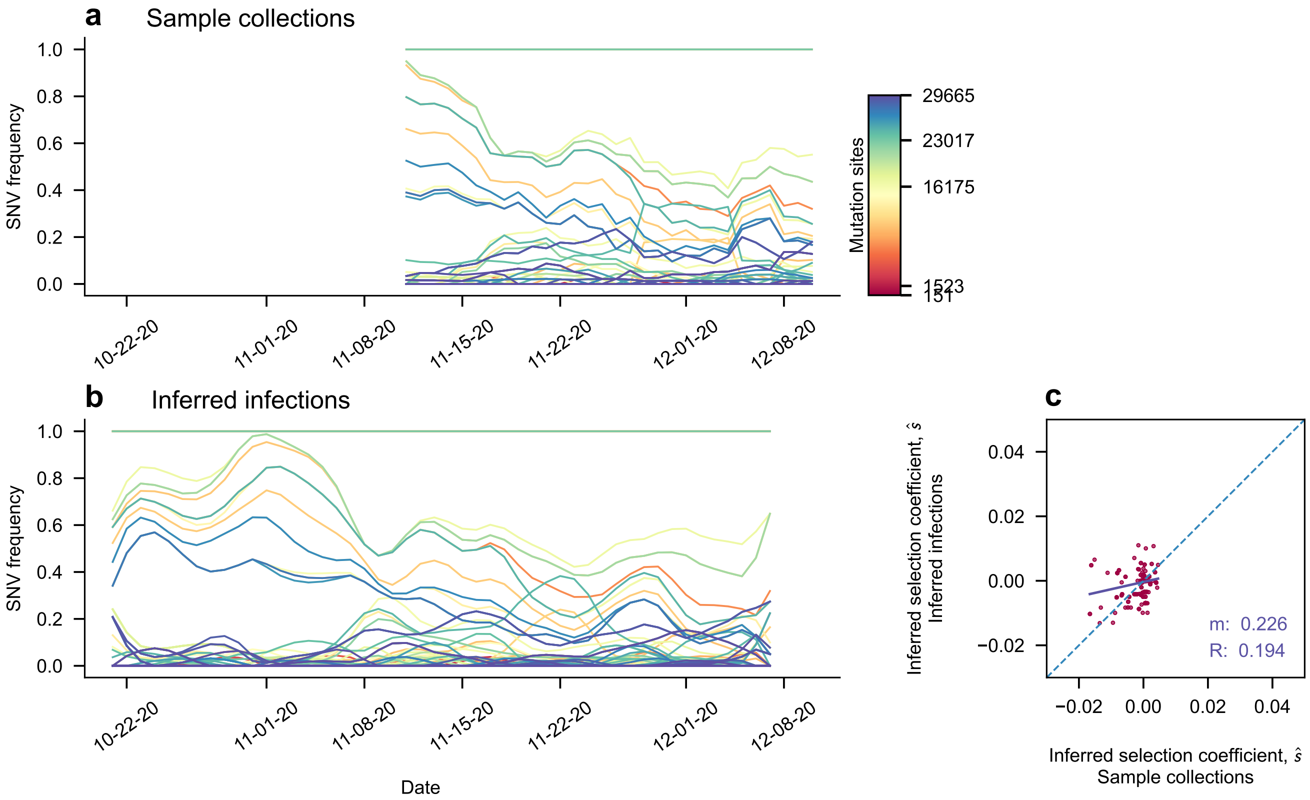

Supplementary Fig. 17. Increased sampling at the beginning and end of an outbreak (Romania). Significant increases in trajectory length with back-projection lead to more moderate correlations between selection coefficients inferred with and without back-projection due to differences in information about mutations driving changes in transmission.

bioRxiv preprint doi: https://doi.org/10.1101/2025.06.29.662219; this version posted July 1, 2025. The copyright holder for this preprint (which was not certified by peer review) is the author/funder, who has granted bioRxiv a license to display the preprint in perpetuity. It is made available under aCC-BY 4.0 International license.

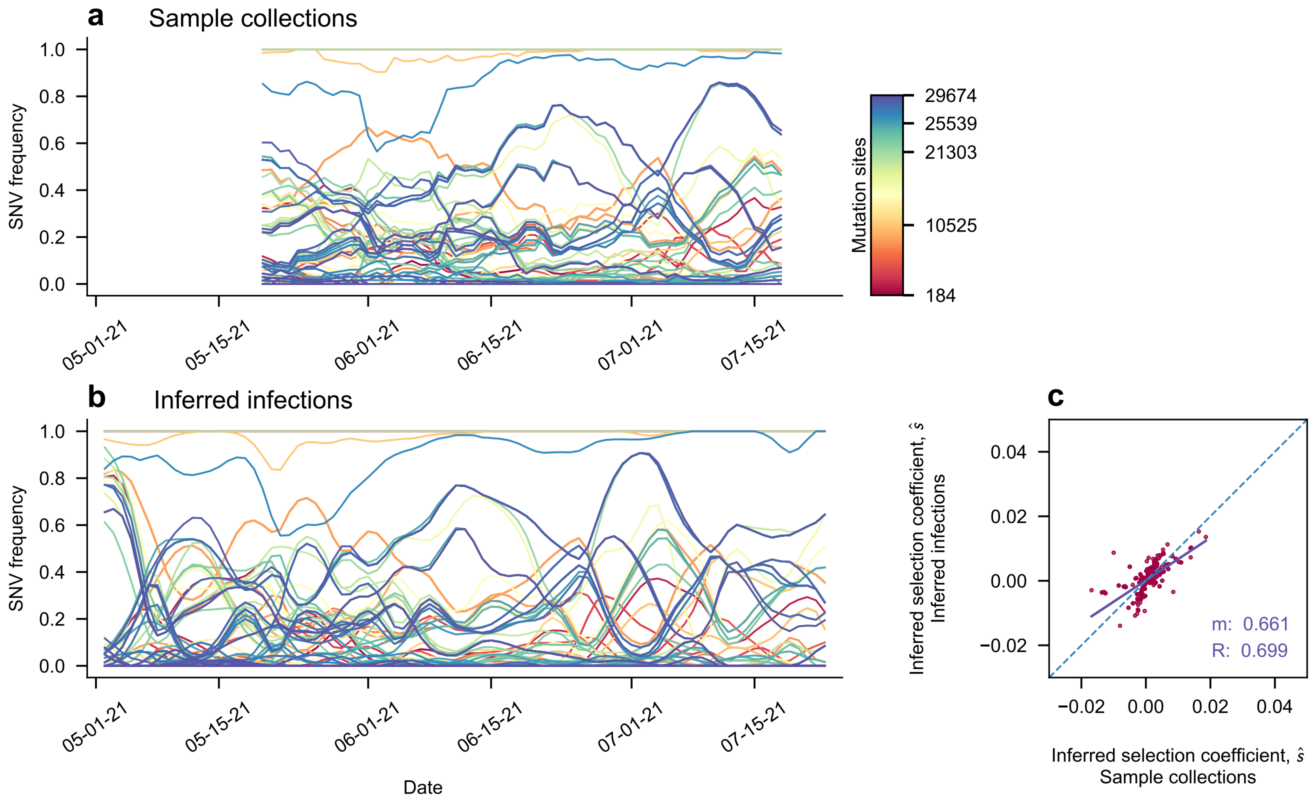

Supplementary Fig. 18. Increased sampling at the beginning and end of an outbreak (Manitoba). Significant increases in trajectory length with back-projection lead to more moderate correlations between selection coefficients inferred with and without back-projection due to differences in information about mutations driving changes in transmission.

bioRxiv preprint doi: https://doi.org/10.1101/2025.06.29.662219; this version posted July 1, 2025. The copyright holder for this preprint (which was not certified by peer review) is the author/funder, who has granted bioRxiv a license to display the preprint in perpetuity. It is made available under aCC-BY 4.0 International license.

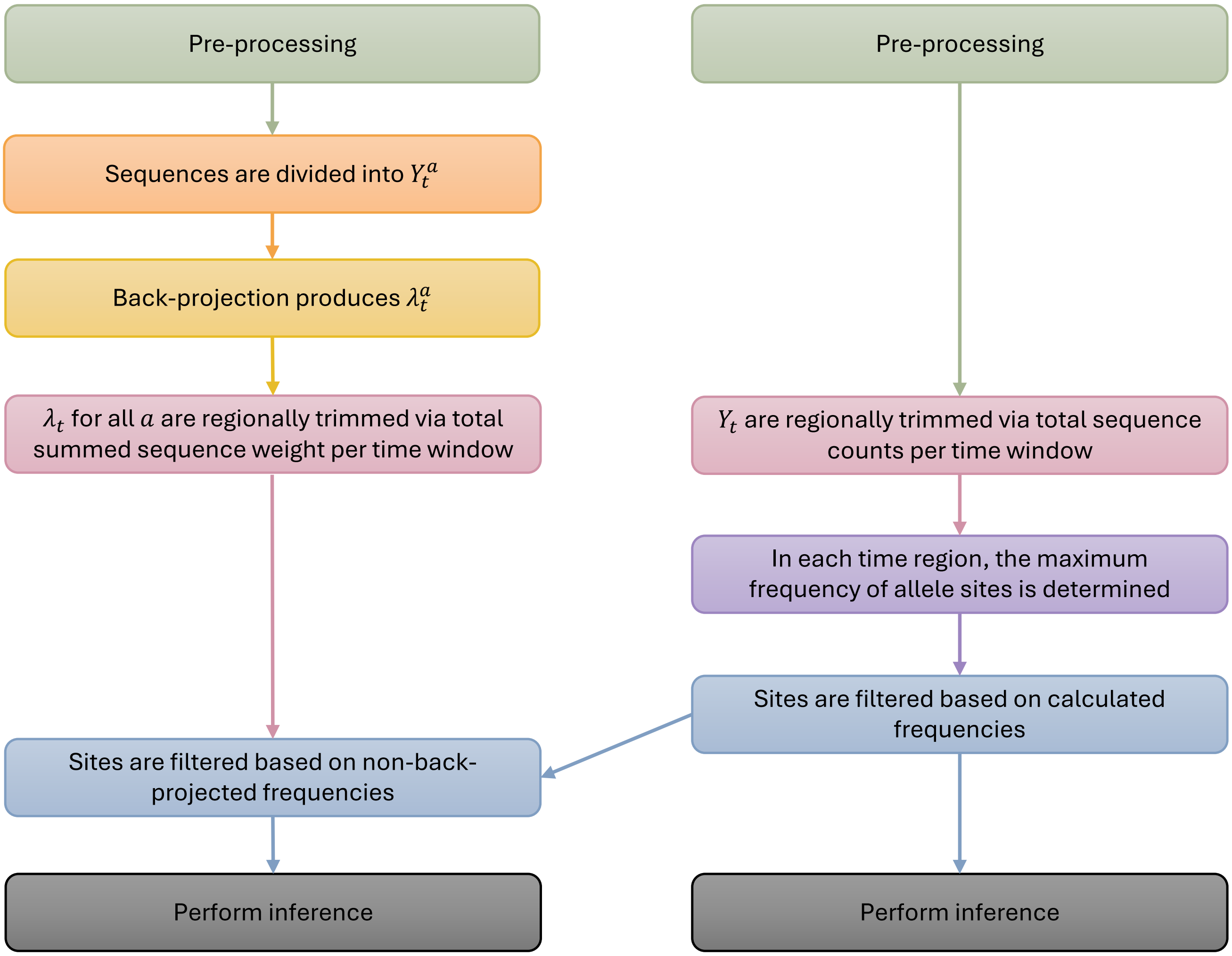

Supplementary Fig. 19. Processing steps with and without back-projection. For comparison, we perform analysis with and without the back projection steps. To allow comparison, some processing steps are performed with the same criteria, as the trimming step, and others are adapted to depend on the parameters in the unprocessed set. In particular, a filtering step chosen to eliminate rare mutations is performed in the unprocessed set, and those same filtered alleles are chosen for comparison in the back-projected dataset. Code for filtering back projected alleles is available, but not used in this analysis.
